# A Rare Case of Emphysematous Gastritis Secondary to Chemotherapy

**DOI:** 10.7759/cureus.18895

**Published:** 2021-10-19

**Authors:** Olisaemeka D Ogbue, Abdo Haddad, Hamed Daw

**Affiliations:** 1 Internal Medicine, Cleveland Clinic Fairview Hospital, Cleveland, USA; 2 Hematology and Oncology, Cleveland Clinic Fairview Hospital, Cleveland, USA

**Keywords:** bevacizumab, fluorouracil, colon adenocarcinoma, chemotherapy, emphysematous gastritis

## Abstract

Emphysematous gastritis is a rare medical condition characterized by the presence of intra-mural air in the stomach associated with portal venous air tracking to a variable degree. There are no established guidelines favoring surgery over medical management. We present a case of a 64-year-old Caucasian male with a history of stage four colon adenocarcinoma with peritoneal carcinomatosis, malignant ascites, and liver metastasis status post-three cycles of chemotherapy who presented to the emergency room with complaints of generalized abdominal pain, nausea, non-bilious vomiting, and melena stools. He was managed conservatively as a case of sepsis secondary to emphysematous gastritis and made a full recovery. To our knowledge, this is the first reported case of emphysematous gastritis in an adult with colon cancer. Although we cannot establish a causal link between his chemotherapy regimen and emphysematous gastritis, the combined effect of an immunosuppressive state caused by advanced malignancy and cytotoxic effects of chemotherapy are the probable risk factors in our patient. We described the possible mechanisms of mucosal disruption by fluorouracil and bevacizumab in our case. Despite historically having a poor prognosis, emphysematous gastritis can be managed conservatively on a case-by-case basis. Clinicians should be aware that chemotherapy can be a predisposing factor to developing this rare condition.

## Introduction

Emphysematous gastritis is a rare medical condition characterized by the presence of intra-mural air in the stomach associated with portal venous air tracking to a variable degree. A severe variant of pneumatosis intestinalis, emphysematous gastritis has been traditionally considered to have mortality rates as high as 60-80% [1]. There are no established guidelines favoring surgery over medical management.

We describe a unique case of emphysematous gastritis occurring during chemotherapy in a patient with advanced colon malignancy. The potential mechanisms of this extremely rare adverse effect of chemotherapy are discussed.

## Case presentation

We present a case of a 64-year-old Caucasian male with a history of stage four colon adenocarcinoma with peritoneal carcinomatosis, malignant ascites, and liver metastasis status post-three cycles of leucovorin, fluorouracil, irinotecan, and bevacizumab (FOLFIRI/Avastin) who presented to the emergency room with complaints of generalized abdominal pain, nausea, non-bilious vomiting, and melena stools.

He had complained of abdominal pain, nausea, and diarrhea two weeks prior at an outpatient oncology clinic while on chemotherapy for which he received diphenoxylate/atropine, antiemetics, and 2 mg dexamethasone. These medications provided only minimal relief. He had the dosing of 5-fluorouracil (5-FU) and irinotecan decreased at the completion of cycle three of the chemotherapy regimen due to these new-onset symptoms.

He then developed new-onset vomiting after two weeks with an increase in severity of abdominal pain and was advised to present to the emergency room by his oncologist out of concern for bowel obstruction.

In the emergency room, he was hypotensive (blood pressure 92/60), tachycardic (heart rate 104) and had diffuse abdominal tenderness with guarding. Laboratory investigations were notable for leukocytosis of 67.62 K/uL, which was neutrophil predominant (neutrophils 98% and absolute neutrophil count of 66.27 K/uL).

**Figure 1:**
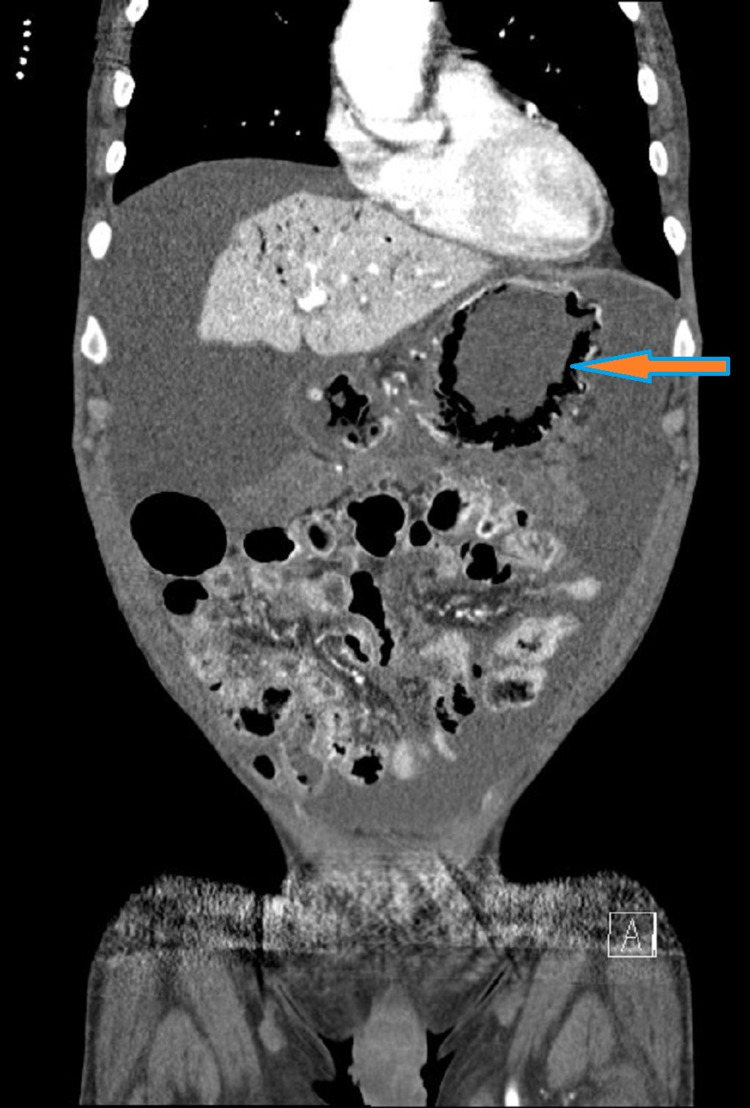
Coronal sectional view of the CT scan of the abdomen showing gas within the wall of the stomach. Arrow demonstrating intra-mural gastric air.

**Figure 2:**
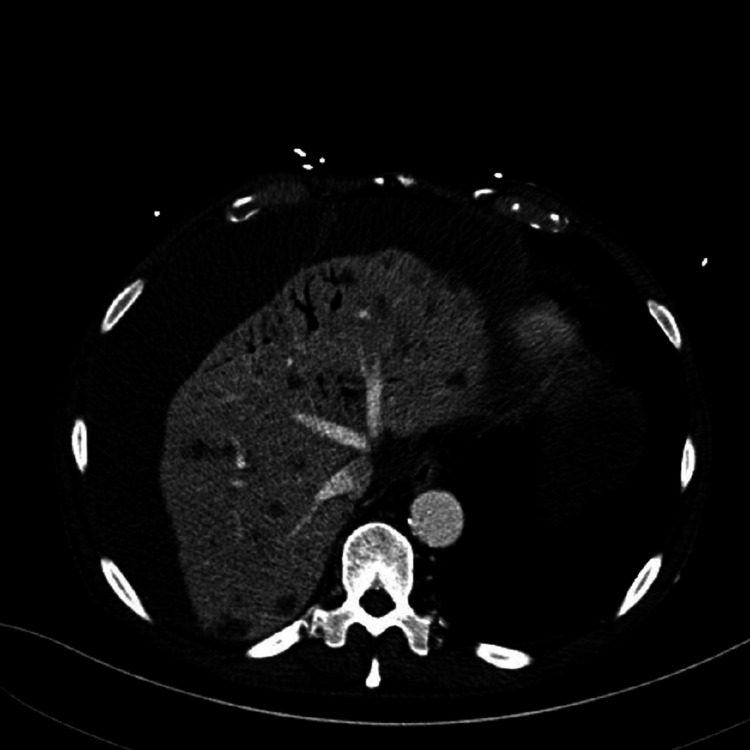
Cross-sectional view of the same contrast-enhanced CT scan of the abdomen demonstrating portal venous air.

These symptoms were concerning for acute abdomen, thus, a CT scan of the abdomen and pelvis was done. Imaging showed emphysematous gastritis with extensive portal gas (Figures 1, 2).

He was managed as a case of sepsis secondary to emphysematous gastritis. Samples for blood and urine cultures were taken. The patient was given intravenous fluids, broad-spectrum antibiotics and was transferred to the surgical intensive care unit in stable condition, where he was managed conservatively. Conservative management included bowel rest, nasogastric decompression, intravenous proton pump inhibitor in addition to empiric antibiotic coverage. Empiric antibiotics comprised of intravenous vancomycin, piperacillin-tazobactam, and fluconazole. This was de-escalated to intravenous ertapenem on receipt of negative blood and urine culture results. Gastroenterology service decided against endoscopy as the patient had improved clinically after one week on conservative treatment. His diet was advanced, and the patient was discharged on a two-week course of intravenous ertapenem. He had made a full recovery but unfortunately, due to cancer progression was transitioned to hospice and died four months later.

## Discussion

Pneumatosis intestinalis is the occurrence of gas within the walls of the gastrointestinal tract. The rarest site of this occurrence is the stomach wall [2]. Emphysematous gastritis and gastric emphysema are distinct entities at opposite ends of the clinical spectrum. They both have radiologic evidence of intra-mural gastric air; the latter has a benign clinical course with no systemic signs. Differences between these two entities are summarized in Table 1.

**Table 1:** Differences between gastric emphysema and emphysematous gastritis.

Etiology	Non-infective source of intra-mural gastric air	Infective source. Gas-forming organisms
Predisposing factors	Gastroenteritis, projectile vomiting, gastric outlet obstruction.	Diabetes, immunosuppression and alcohol abuse.
Presentation	Usually asymptomatic and hemodynamically stable.	Abdominal pain, hematemesis, melena, sepsis, and septic shock.
Imaging	Linear pattern with no wall thickening or portal venous air.	CT usually shows streaky and linear pattern of air in gastric wall. Associated air in portal venous system.
Clinical course	Benign, usually self-limiting.	Requires medical or surgical management, often fatal.

The gastric mucosal barrier is relatively resistant to infection due to its acidity and rich blood supply. In emphysematous gastritis, gas producing organisms invade the gastric mucosa resulting in a systemic inflammatory response. Organisms commonly implicated include *Streptococcus* *species*, *Escherichia coli*, *Enterobacter species*, *Clostridium species*, *Staphylococcus aureus*, *Klebsiella pneumoniae*, *Pseudomonas aeruginosa*, and *Candida species*. In 42.4% of cases, however, no organism is identified [3]. The exact mechanism of mucosal disruption and subsequent microbial invasion is often unknown; however, several predisposing factors have been described in the literature including diabetes, renal failure, leukemia, adenocarcinoma, immunosuppression, peptic ulcer disease, alcohol abuse, ingestion of foreign/corrosive materials, gastrostomy feeding, intra-abdominal surgery, and medications such as long-term steroids and non-steroidal anti-inflammatory drugs.

There have been less than 100 cases of emphysematous gastritis reported in existing literature in the English language. Pneumatosis intestinalis has been associated with chemotherapeutic agents such as fluorouracil, docetaxel, and cisplatin but emphysematous gastritis due to chemotherapy is extremely rare. There has been a case reported in a child with leukemia on chemotherapy and steroids [4]. To our knowledge, this is the first reported case of emphysematous gastritis in an adult with colon cancer. Although we cannot establish a causal link between his chemotherapy regimen (FOLFIRI/Avastin) and emphysematous gastritis, the combined effect of an immunosuppressive state caused by advanced malignancy and cytotoxic effects of chemotherapy are the probable risk factors in our patient. 5-FU, especially when administered with leucovorin is more commonly associated with large bowel toxicity. Small bowel and stomach toxicities are less commonly reported. A potential mechanism of mucosal disruption is stomach ulceration and alteration of mucosal blood flow caused by 5-FU. Fata et al. [5] described a group of six patients with adenocarcinoma of the colon, treated with 5-FU and leucovorin who developed extensive small bowel ulceration with no involvement of the colon. They propose that the local effects of 5-FU on the mucosal blood flow or thrombogenic and angiospastic effects on the vascular endothelium are responsible for small bowel ulceration in these patients.

Avastin (bevacizumab) is a monoclonal antibody targeting vascular endothelial growth factor (VEGF). It is an anti-angiogenic drug that has been associated with gastric perforation [6]. Mechanisms of bowel perforation that have been suggested are attributed to the anti-VEGF effects compromising bowel wall integrity, impaired healing of bowel injury, and ischemia related to mesenteric thrombosis [7].

**Figure 3:**
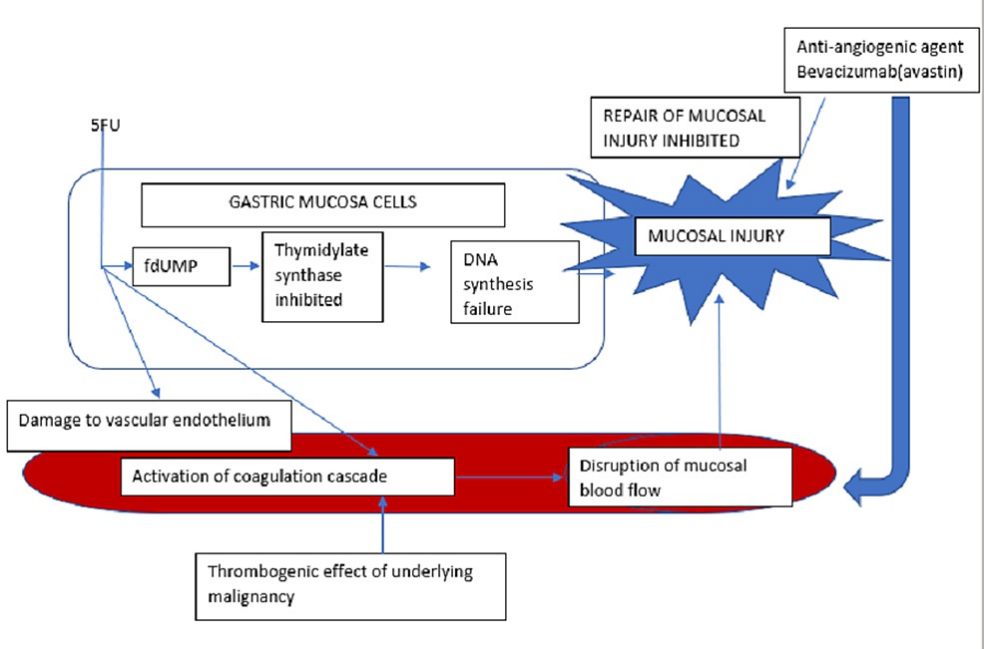
Mechanism of mucosal injury from effects of chemotherapy and malignancy. fdUMP: fluorordeoxyuridylate, 5-FU: 5-fluorouracil.

The mechanisms of action of chemotherapy agents that cause mucosal injury are described in Figure 3. The resultant mucosal disruption can serve as a nidus of infection for gas-forming organisms resulting in emphysematous gastritis.

Our patient presented with septic shock and radiologic features of portal venous gas but had no signs of peritonitis and thus was managed conservatively with bowel rest, nasogastric decompression, intravenous proton pump inhibitor, and broad-spectrum antibiotics. He had negative blood and urine cultures and this was the rationale for broad-spectrum coverage in our case. Early antibiotic administration has been associated with better mortality outcomes [8]. Conservative management is agreeable with current trends, which favor medical management of emphysematous gastritis. It has been observed that after the year 2000, fewer patients with emphysematous gastritis underwent surgical intervention compared to prior (62.5% before 2000 versus 22.2% after 2000, P = 0.002), and this was associated with a lower mortality rate (59.4% before 2000 versus 33.3% after 2000, P = 0.046) [3]. The role of endoscopy is not established as the diagnosis of emphysematous gastritis can be based on clinical and radiologic findings. Endoscopy, however, may be useful to identify the causative organism(s) from gastric tissue biopsy/culture to guide antibiotic therapy when blood cultures are negative.

## Conclusions

In conclusion, we present a case of emphysematous gastritis occurring during chemotherapy. We described the possible mechanisms of mucosal disruption by 5-FU and bevacizumab in our case. Despite the historically poor prognosis, emphysematous gastritis can be managed conservatively on a case-by-case basis. Clinicians should be aware that chemotherapy can be a predisposing factor to developing this rare condition.

## References

[1] Szuchmacher M, Bedford T, Sukharamwala P, Nukala M, Parikh N, Devito P (2013). Is surgical intervention avoidable in cases of emphysematous gastritis? A case presentation and literature review. Int J Surg Case Rep.

[2] Majumder S, Trikudanathan G, Moezardalan K, Cappa J (2012). Vomiting-induced gastric emphysema: a rare self-limiting condition. Am J Med Sci.

[3] Nasser H, Ivanics T, Leonard-Murali S, Shakaroun D, Woodward A (2019). Emphysematous gastritis: a case series of three patients managed conservatively. Int J Surg Case Rep.

[4] Rowen M, Myers M, Williamson RA (1976). Emphysematous gastritis in a leukemic child. Med Pediatr Oncol.

[5] Fata F, Ron IG, Kemeny N, O'Reilly E, Klimstra D, Kelsen DP (1999). 5-Fluorouracil-induced small bowel toxicity in patients with colorectal carcinoma. Cancer.

[6] Turco LC, Ferrandina G, Vargiu V (2020). Extreme complications related to bevacizumab use in the treatment of ovarian cancer: a case series from a III level referral centre and review of the literature. Ann Transl Med.

[7] Shinagare AB, Howard SA, Krajewski KM, Zukotynski KA, Jagannathan JP, Ramaiya NH (2012). Pneumatosis intestinalis and bowel perforation associated with molecular targeted therapy: an emerging problem and the role of radiologists in its management. AJR Am J Roentgenol.

[8] Elson MZ, Monzón LL, Orta JE, Fierro PC, Monteverde VV, Arbeloa CS (2016). Emphysematous gastritis: effectiveness of early antibiotic therapy. Gastroenterol Hepatol.

